# N-Acetylcysteine and Allopurinol Confer Synergy in Attenuating Myocardial Ischemia Injury via Restoring HIF-1α/HO-1 Signaling in Diabetic Rats

**DOI:** 10.1371/journal.pone.0068949

**Published:** 2013-07-18

**Authors:** Xiaowen Mao, Tingting Wang, Yanan Liu, Michael G. Irwin, Jing-song Ou, Xiao-long Liao, Xia Gao, Yuan Xu, Kwok F. J. Ng, Paul M. Vanhoutte, Zhengyuan Xia

**Affiliations:** 1 Department of Anaesthesiology, University of Hong Kong, Hong Kong, China; 2 Division of Cardiac Surgery, The First Affiliated Hospital, Sun Yat-sen University, Guangzhou, China; 3 Department of Endocrinology, Beijing Chaoyang Hospital, Capital Medical University, Beijing, China; 4 Department of Pharmacology and Pharmacy, University of Hong Kong, Hong Kong, China; 5 Shenzhen Institute of Research and Innovation, The University of Hong Kong, Shenzhen, China; Virginia Commonwealth University, United States of America

## Abstract

**Objectives:**

To determine whether or not the antioxidants N-acetylcysteine (NAC) and allopurinol (ALP) confer synergistic cardioprotection against myocardial ischemia/reperfusion (MI/R) injury by stabilizing hypoxia inducible factor 1α (HIF-1α)/heme oxygenase 1 (HO-1) signaling in diabetic myocardium.

**Methods:**

Control or diabetic [streptozotocin (STZ)-induced] Sprague Dawley rats received vehicle or NAC, ALP or their combination for four weeks starting one week after STZ injection. The animals were then subjected to thirty minutes of coronary artery occlusion followed by two hours reperfusion in the absence or presence of the selective HO-1 inhibitor, tin protoporphyrin-IX (SnPP-IX) or the HIF-1α inhibitor 2-Methoxyestradiol (2ME2). Cardiomyocytes exposed to high glucose were subjected to hypoxia/re-oxygenation in the presence or absence of HIF-1α and HO-1 achieved by gene knock-down with related siRNAs.

**Results:**

Myocardial and plasma levels of 15-F2t-isoprostane, an index of oxidative stress, were significantly increased in diabetic rats while cardiac HO-1 protein and activity were reduced; this was accompanied with reduced cardiac protein levels of HIF-1α, and increased post-ischemic myocardial infarct size and cellular injury. NAC and ALP given alone and in particular their combination normalized cardiac levels of HO-1 and HIF-1α protein expression and prevented the increase in 15-F2t-isoprostane, resulting in significantly attenuated post-ischemic myocardial infarction. NAC and ALP also attenuated high glucose-induced post-hypoxic cardiomyocyte death *in vitro*. However, all the above protective effects of NAC and ALP were cancelled either by inhibition of HO-1 or HIF-1α with SnPP-IX and 2ME2 *in vivo* or by HO-1 or HIF-1α gene knock-down *in vitro*.

**Conclusion:**

NAC and ALP confer synergistic cardioprotection in diabetes via restoration of cardiac HIF-1α and HO-1 signaling.

## Introduction

Myocardial infarction is a major perioperative complication that is associated with significant mobility and mortality, particularly in patients with diabetes. Reperfusion therapies restore coronary flow, but reperfusion may itself cause lethal tissue injury termed ischemia reperfusion injury (IRI) [1]. The mortality is higher in patients with diabetes after acute myocardial ischemia with or without coronary artery bypass grafting surgery to restore blood perfusion [2], [3]. Excessive production of reactive oxygen species (ROS) and the subsequent increase in oxidative stress under high glucose conditions plays a critical role in this pathology [4].

Stabilization of hypoxia-inducible factor-1 (HIF) improves ventricular performance, remodeling, and vascularization after myocardial infarction in rats [5]. HIF is a key regulator in response to low-oxygen (O_2_) conditions (hypoxia/anoxia) [6]. It is a heterodimer consisting of an inducible α subunit and a constitutively expressed β subunit; its activity is determined by stable expression of the α-subunit [7]. HIF-1α is increased in the euglycemic heart in response to ischemia, while HIF-1α -mediated cellular responses to hypoxia are blunted in hyperglycemic rats [8], [9], but the underlying mechanism is unclear. Heme oxygenase-1 (HO-1) is a down-stream protein of HIF-1α and plays a key role in protecting the heart against ischemic injury [10]. Inhibition of HO-1 gene expression [11] or HO-1 gene knock-down [12] exacerbates while HO-1 gene overexpression [13], [14] attenuates myocardial infarction and ventricular arrhythmias after myocardial ischemia and reperfusion in mice. Induction of HO-1 occurs as an adaptive defensive mechanism in response to a variety of stimuli including oxidative stress and hypoxia [15], [16] while HO-1 is reduced in the diabetic myocardium after ischemia reperfusion injury [17]. HIF-1α mediated up-regulation of HO-1 is beneficial to the ischemic myocardium in non-diabetic rodents [10], but related information under diabetic conditions is lacking. Thus, it is possible that treatments that enhance myocardial HIF-1α/HO-1 expression in response to ischemia reperfusion under hyperglycemic conditions should confer cardioprotection in diabetes.

HIF-1α production is decreased under oxidative conditions [18] and the deficiency in HIF-1α results in increased myocardial damage in response to ischemic insult [19]. In diabetes, the high glucose levels favor the production of ROS as a result of glucose auto-oxidation, metabolism and the development of advanced glycosylation end products [4]. Superoxide dismutase (SOD) is a major endogenous antioxidant enzyme but its ROS scavenging ability is decreased under hyperglycemic conditions [20]. Likewise, the reduced HIF-1α expression in the diabetic myocardium is associated with decreased tolerability to myocardial ischemic insult [8], [21]. However, the link between hyperglycemia induced increases in oxidative stress and decreases in myocardial HIF-1α in the context of myocardial ischemia-reperfusion has not been elucidated. The antioxidants N-acetylsysteine (NAC) and allopurinol (ALP), when applied individually at a dose that is sufficient to completely prevent hyperglycemia-induced oxidative stress in diabetic rats, each moderately reduced post-ischemic myocardial infarction, while their combined administration profoundly reduced infarct size [22] without further reduction in either cardiac or systemic levels of 15-F2t isoprostane, a specific index of ROS-induced oxidative stress [23]. This finding suggests that NAC and ALP may have exerted synergistic cardioprotection via mechanisms not limited to boosting antioxidant capacity. Given that HIF-1α deficiency exacerbates myocardial IRI and that cardiac HIF-1α is decreased in diabetes [24], we postulated that reduction in myocardial HIF-1α expression as a result of high-glucose induced oxidative stress may be a major mechanism explaining why diabetic hearts are less resistant to IRI than those from non-diabetic subjects. Further, we hypothesized that enhancement of endogenous myocardial HIF-1α is a key mechanism whereby antioxidants NAC and ALP confer synergistic cardioprotection against myocardial IRI in diabetes. This hypothesis was tested *in vivo* in type 1 diabetic rats and in isolated adult rat cardiomyocytes and H9C2 cell line subjected to hypoxia/re-oxygenation *in vitro*.

## Materials and Methods

### In Vivo Studies

#### Experimental animals

Male adult Sprague-Dawley with streptozotocin (STZ)-induced diabetes and age-matched non-diabetic control rats were used. All rats (250±10 g, 6–8 weeks) were obtained from and housed in the Laboratory Animal Service Center (University of Hong Kong) and received standard care in accordance with the principles of Animal Care of the University of Hong Kong. The committee on the Use of Live Animals in Teaching and Research (CULATR) approved the experimental protocols.

#### Induction of diabetes and antioxidant treatment

**Figure 1:**
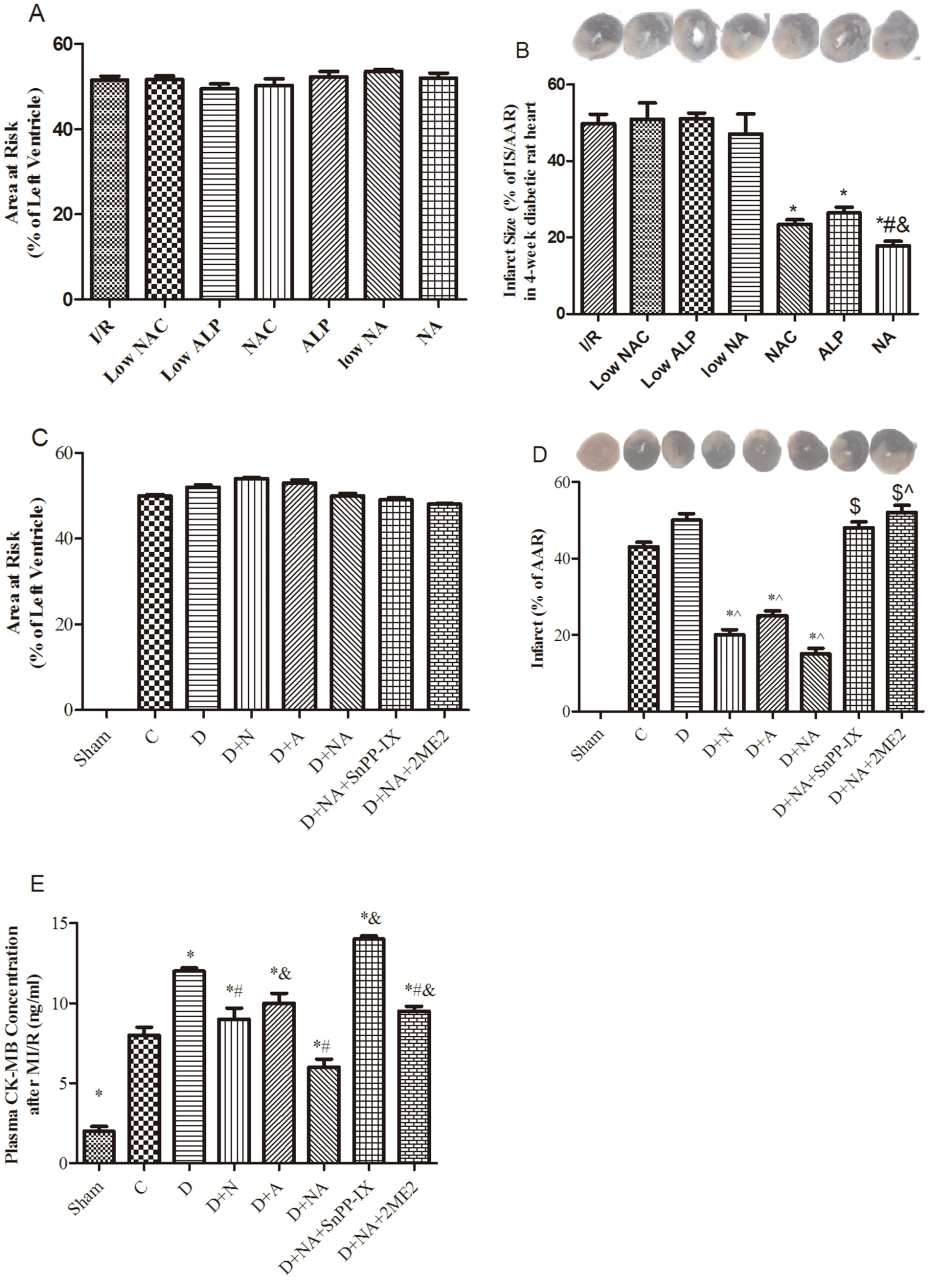
Myocardial ischemia reperfusion injury assessed after 30 minutes of ischemia followed by two hours of reperfusion in control and diabetic rats with or without treatment. Statistical analysis of the ratio of the area at risk (AAR)/left ventricle (LV) (A, C) and percent infarct size (IS) expressed as a ratio to the AAR (B, D) and plasma CK-MB levels (E). In a preliminary dose-finding study (A, B), rats were treated with low doses of N-acetylcysteine (NAC) (0.5 g/kg/day, Low NAC) or allopurinol (ALP) (50 mg/kg/day, Low ALP) or their combination (Low NA), or relatively higher doses of NAC (1.5 g/kg/day) or ALP (100 mg/kg/day) or their combination (NA), for a duration of 4 weeks before being subjected to myocardial ischemia reperfusion. Data are mean ± S.E.M. (n = 4–5 per group), *P<0.01 vs. I/R group, ^#^P<0.01 vs. NAC group and ^&^P<0.01 vs. ALP group. Low NAC plus low ALP only moderately (P>0.1 vs. group I/R) attenuated post-ischemic myocardial IS. Therefore, the subsequent studies were performed in rats treated with high doses of NAC and/or ALP (C–E). Sham, C and D indicate sham operation, control or diabetes respectively. D+N, D+A, D+NA, D+NA +SnPP-IX, D+NA+2ME2 indicate control or diabetic rats respectively treated with NAC (N), ALP (A), NAC+ALP (NA) or NA+SnPP-IX or NA +2ME2. Data are expressed as mean ± S.E.M. (n = 7). ^?^P<0.05 or P<0.01 vs. C; ^*^P<0.05 or P<0.01 vs. D, ^$^P<0.05, or P<0.01 vs. D+NA.

Type 1 diabetes was induced as described [22]. Some of the diabetic rats were treated with NAC or ALP (Sigma-Aldrich, St. Louis, MO, USA) alone or in combination. The duration of the treatment was four weeks, starting one week after induction of diabetes. The optimal dosages of NAC and ALP were selected according to our preliminary dose-finding experiment. We started with relatively low dosages of NAC (0.5 g/kg/day) and ALP (50 mg/kg/day) as used respectively in the studies of Fiordaliso *et al* [25] and Xiao *et al* [26]. NAC when given at 0.5 g/kg/day for 3 months could partially attenuate hyperglycemia-induced myocardial oxidative stress and cardiac cell death [25] in diabetic rats, while ALP when given at 50 mg/kg/day for 4 weeks did not significantly reduce post-ischemic myocardial infarction despite that it could partially reduce myocardial ROS production [26]. Since these two antioxidants targeting different components of ROS [27], [28], we explored whether or not the combinational use of NAC at 0.5 g/kg/day (low dose NAC) and ALP at 50 mg/kg/day (low dose ALP) could confer cardioprotection against MI/R, and compared with the effects of relatively higher doses of NAC (1.5 g/kg/day [22], [29] ) and/or ALP (100 mg/kg/day [22] [30]). STZ-induced diabetic rats were treated for 4 weeks with the respective antioxidants above-mentioned starting at 1 week after the induction of diabetes, before being subjected to 30 minutes coronary ligation followed by two hours of reperfusion. We found that neither low-dose NAC nor low-dose ALP, alone or in combination could significantly reduce post-ischemic myocardial infarction in diabetic rats while NAC (1.5 g/kg/day) and ALP (100 mg/kg/day) conferred synergy in reducing post-ischemic myocardial infarction (Fig. 1A and B). Base on the above dose-finding studies, NAC at 1.5 g/kg/day and ALP at 100 mg/kg/day were used the subsequent experiments. Blood samples were withdrawn from the inferior vena cava, and plasma was extracted and stored at −80°C until being analysis. The left ventricle was removed and rinsed with ice-cold phosphate buffered saline, and weighed. Subgroups of rats were subjected to myocardial ischemia reperfusion (MI/R) as described [22].

#### Effects of NAC and ALP on MI/R in STZ-induced diabetes

The diabetic and the age-matched normal rats were randomly divided into five groups of seven rats each: Control group(C), untreated diabetic rats (D), diabetic rats treated with NAC (D+N) or ALP (D+A) alone or in combination (D+NA). Previous studies show that at the doses used, NAC and ALP do not significantly affect myocardial levels of the lipid peroxidation product 15-F2t-isoprostane in control rats [31]. Subsequently, ALP and NAC were only given to diabetic rats in the current study. The rats were subjected to coronary ligation as described below.

#### Effects of HIF-1 α/HO-1 inhibition

Six groups of seven rats each [sham-operated (Sham group), control(C group), diabetic (D group), diabetics treated with NAC plus ALP (D+NA group) diabetic treated with NAC plus ALP concomitant administrations of the HIF-1α blocker 2-methoxyestradiol (D+NA+2ME2 group), or diabetic treated with NAC plus ALP and the HO-1 blocker () (D+ NA+SnPP-IX group)] were subjected to *in vivo* coronary ligation as described above. 2-Methoxyestradiol (2ME2; Sigma-Aldrich, St. Louis, MO, USA) was administrated at15 mg/kg intraperiteonally [32] before inducing ischemia. Tin protoporphyrin-IX (SnPP-IX; Sigma-Aldrich) was given at 0.7 mg/kg [33] intravenously for two days before inducing coronary ischemia.

#### Echocardiography

M-mode echocardiographic studies were performed at termination using a 17.5 MHz liner array transducer system (Vevo 770TM High Resolution Imaging System; Visual Sonics, Toronto, Canada) as described [34]. Left ventricular contractile and diastolic function parameters were measured and calculated as described [31], [34]. Three representative cardiac cycles were recorded and averaged for each measurement.

#### *In vivo* coronary ligation model and infarct size (IS) determination

Rats were subjected to 30 minutes of left anterior descending artery (LAD) ligation followed by two hours of reperfusion as described [22]. The *in vivo* MI/R model of 30 minutes LAD occlusion and two hours of reperfusion was chosen based on preliminary studies which showed that the majority of the diabetic rats did not survive 60 minutes of LAD occlusion. Likewise, although the diabetic rats could withstand 45 minutes of LAD occlusion, the resulting post-ischemic myocardial infarct size was much higher than that in the control rats (n = 4–5/group, data not shown) and hence the diabetic animals subjected to 45 minutes of LAD occlusion did not survive for two hours during post-ischemic reperfusion. Hemodynamics was continuously monitored using PowerLab monitoring system (ML750 PowerLab/4 sp with MLT0380 Reusable BP Transducer; AD Instruments, CO Springs, CO, USA). Myocardial infarct size (IS) was measured using TTC (1% 2, 3, 5-triphenyltetrazolium chloride) staining as described [35]. IS was expressed as a percentage of the area at risk (AAR). Creatinine kinase-MB (CK-MB) isoenzyme is a major biomarker for myocardial cellular injury and CK-MB content was measured using a commercial kit (Uscn Life Science Inc. Wuhan, China) in blood samples collected after two hours reperfusion.

#### *In situ* apoptotic cell death detection

TdT-mediated DUTP-X nick end labeling (TUNEL stain) was employed to detect apoptotic cell death according to the manufacturer’s instruction (Roche Applied Science, Indianapolis, IN, USA). Briefly, paraffin embedded left ventricular tissue was sliced (5-µm thick sections) and deparaffinized. Subsequently, the sections were permeabilized using proteinase K (30 µg/ml, 30 minutes, 37°C) and were washed in phosphate buffered saline (PBS). The sections were then incubated with TUNEL reaction mixture for 60 minutes and washed twice in PBS. Sections were incubated with anti-fluorescein POD followed by the addition of DAB and examined under a light microscope. As a positive control, DNase I was used to induce DNA strand breaks. For the negative control, TdT was omitted from the reaction mixture. The number of TUNEL-positive cardiac myocytes (which were stained brown) was determined by counting them within defined areas (30×30 grids) of each slide and the average of ten histological sections was reported as means ± S.D. All morphometric measurements were carried out by two independent expert examiners in a blinded manner and results of the treated groups were expressed in comparison to the control group.

#### Plasma and cardiac free 15-F2t-IsoP and SOD measurement

Free 15-F2t-IsoP, a specific marker of oxidative stress *in vivo*, was measured by using an enzyme immunoassay kit (Cayman Chemical, Ann Arbor, MI, USA) as described [27]. The value of plasma free 15-F2t-IsoP was expressed as pg/mL in plasma. Myocardial SOD activity was detected in cardiac tissue homogenates using commercially available kits (Cayman Chemical) as described previously [36]. Myocardial reduced/oxidized glutathione couple (GSH/GSSG) ratio was measured in cardiac tissue also using a commercially available assay kit (Sigma-Aldrich) according to the manufacturer’s protocol.

### In Vitro Studies

#### Isolation of adult rat cardiomyocytes and hypoxia/re-oxygenation (H/R)

Isolated primarily cultured adult rat cardiomyocytes were prepared as described [22]. The cells were treated with high glucose (25 mM glucose in the culture medium) to mimic the diabetic state for 48 hours before being subjected to H/R. Hypoxic conditions were achieved by equilibrating a humidified chamber containing myocytes with 95% N_2_ and 5% CO_2_ via a gas transfusion apparatus (BioSpherix, Redfield, NY, USA) and hypoxia was confirmed by demonstrating a chamber O_2_ concentration not higher than 0.1%. After 45 minutes of hypoxia, the cells were transferred to room air (CO_2_ incubator) to achieve re-oxygenation. After 120 minutes of re-oxygenation, the cells and the medium were collected and stored at −80°C until analysis.

#### Effects of HIF-1α/HO-1 inhibition

The primarily cultured adult rat cardiomyocytes were randomly divided into six groups: non-H/R control (C), H/R control (H/R), high glucose (25 mM) medium (H-G), H-G treated with 1 mM NAC (H-G+N) or with 100 µM ALP (H-G+A), with NAC plus ALP(H-G+NA), with NAC plus ALP in the presence of 2ME2 (H-G+NA+2ME2) 5 µM [37] or with NAC plus ALP in the presence of SnPP-IX(H-G+ NA+ SnPP-IX)10 µM [38]. Cells were transferred to freshly prepared culture medium with or without inhibitors for 45 minutes before being subjected to 45 minutes of hypoxia and two hours of re-oxygenation. The concentrations of NAC and ALP used were selected based on previous studies [39] [40], and the results of preliminary cell culture experiments indicating that NAC did not confer cellular protection against H/R injury at either low concentrations (0.1 mM to 0.3 mM) or at 3 mM, while that ALP conferred cellular protection at 100 µM but not at 10 to 30 µM or at 300 µM(data not shown). In addition, concomitant administration of NAC and ALP at low (0.3 mM NAC with 30 µM ALP) or at high (3 mM NAC with 300 µM) concentrations did not confer significant cellular protection (data not shown).

#### Transfection and siRNA knockdown in H9C2 cells

Gene silencing was performed using HIF-1 α siRNA, HO-1 siRNA, control siRNA and transfection reagent, purchased from Santa Cruz Biotechnology, Dallas, Texas, USA. Transfection of H9C2 cells was performed following the manufacturer’s instructions. Briefly, 2×10^5^ H9C2 cells were seeded in a six well tissue culture plate in 2 ml of antibiotic-free normal growth the cells were allowed to reach 60–80% confluence. They then were transfected with targeted siRNA and control siRNA. Six hours after the transfection, normal growth medium was added and the cells were incubated at 37°C in a CO_2_ incubator for 24 hours. Then, the cells randomly divided into six groups: control, high-glucose (25 mM), high-glucose and NAC (1 mM) plus ALP (100 µM), high-glucose and NAC plus ALP treated with control siRNA, high-glucose and NAC plus ALP treated with HIF-1α siRNA, and high-glucose and NAC plus ALP treated with HO-1 siRNA. Two days after the treatment, the cells were challenged with 45 min hypoxia-120 min re-oxygenation and used for Western blotting or lactate dehydrogenase (LDH) determination.

#### Cell viability and lactate dehydrogenase (LDH) activity

Cardiomyocyte viability was determined by measuring 3-(4, 5- Dimethylthiazol-2-yl)-2, 5-diphenyltetrazolium bromide (MTT) reduction ability of cardiomyocytes as described [22]. LDH content was measured by LDH Cytotoxicity Assay Kit (Cayman chemical,) using culture medium as described [41].

#### HIF-1α/HO-1 protein expression and HO-1 activity

Frozen ventricular tissue samples were homogenized in lysis buffer. For the culture experiments, the cells were removed upon completion of the incubation period, immediately mixed with cell lysis buffer and then sonicated for ten seconds. The samples were processed for Western blotting analysis following standard methodology as described [22]. Primary antibody of HO-1 (Cell Signaling Technology, Beverly, MA) 1∶500; HIF-1α (Abcam Biochemicals, Cambridge, MA) and GAPDH (Cell Signaling Technology) 1∶1000 were used. Protein bands were detected by a standard ECL method and images were measured by a densitometer with analysis software. Myocardium HO-1 activity was measured by spectrophotometric determination of bilirubin formation according to the manufacturer’s instructions (Genmed Scientifics, Arlington, MA, USA). HO-1 activity was calculated as percentage of control.

#### Mitochondrial membrane potential and apoptosis

Primary cultured cardiomyocytes and H9C2 cells were cultured and treated as described above. After hypoxia/re-oxygenation, the cardiomyocyte mitochondrial potential (MMP) was checked using a JC-1 mitochondrial membrane potential ELISA kit (Cayman Chemical) according to the manufacturer’s protocol. JC-1 stained cells were analyzed using a Live Cell Microscope (Nikon, Melville, New York, USA) (magnification, ×10) and MMP loss was checked using a luminometer (GloMax; Promega, Madison, Wisconsin, USA) and expressed as the percentage of green cells in the total cells. Scale bar: 100 µm.

Cytosolic cytochrome c, Mn SOD and caspase3 protein expression were measured in primary cultured cardiomyocyte and in H9C2 cell by Western blotting as described [42], [43]. Cytosolic proteins were isolated as described [44]. Primary antibodies for cytochrome c, caspase3, cleaved caspase3 and Mn SOD were purchased from Cell Signaling (Cell Signaling).

### Statistical analysis

All values are expressed as means ± standard error of the mean (S.E.M.). One-way analysis of variance (ANOVA) was used for statistical analyses (GraphPad Prism, USA) of data obtained within the same group and between groups, respectively, followed by Tukey’s test for multiple comparisons of group means. *P* values less than 0.05 were considered to indicate statistically significant differences.

## Results

### General Characteristics and Plasma Glucose before Inducing MI/R

STZ-injected rats had significant diabetic symptoms of hyperglycemia, polydipsia, polyphagia and weight loss. The plasma glucose level, water intake and food consumption of the diabetic rats increased but their body weight decreased compared to age-matched normal rats (all P<0.05 D vs. C). NAC and ALP increased water intake and food consumption (all P<0.05 D+NA vs. D). NAC and/or ALP treatment had no significant effect on body weight in diabetic rats. But in combination they reduced the heart/body ratio that was elevated in diabetic rats (P<0.05 D+NA vs. D; P<0.05 D vs. C). Plasma glucose was elevated in the diabetic group as compared to controls. NAC and ALP given alone or in combination had no significant impact on plasma glucose (table 1).

**Table 1:** Effect of N-acetylcysteine and allopurinol on general characteristics in controls and rats with STZ- induced diabetes.

Parameters	C	D	D+N	D+A	D+NA
Water intake (ml/kg/day)	120.6±2.5	790.4±11.2^a^	415.5±7.2^ab^	679.6±10^ab^	455.3±7.3^ab^
Food consumption (g/kg/day)	65.5±4.5	180.1±12.3^a^	135.6±13.3^ab^	179.7±8.5^a^	159.8±8.1^ab^
Body weight (g)	455.7±13.3	320.9±8.5^a^	299.1±10.1^a^	289.6±11.6^a^	291.6±18.5^a^
Plasma glucose (mM)	6.1±0.3	29.3±1.5^a^	29.3±1.0^a^	26.9±1.1^a^	27.9±1.1^a^
Heart/Body weight ratio (g/kg)	3.3±0.2	4.6±0.5^a^	3.5±0.2^b^	4.0±0.1	3.8±0.1^b^

Data are expressed as means± S.E.M. (n = 7). Water intake, food consumption are the average value of four weeks. Body weight, plasma glucose are measured at termination of administration. Control (C), diabetes (D), N- acetylcysteine (NAC), allopurinol (ALP),

a p<0.05 vs. C;

b p<0.05 vs. D.

### NAC and ALP Attenuated Myocardial Injury in Diabetes after MI/R

As shown in Fig. 1C and D, the cardiac area at risk did not differ among groups, while MI/R resulted in moderate increase in myocardial IS in diabetic rats (P>0.05 D. vs. C). NAC and ALP reduced IS individually, while their combination yielded a synergistic effect and further decreased IS in diabetic rats. SnPP-IX or 2ME2 abolished the effect of NAC and ALP in reducing IS. Post-ischemic plasma CK-MB levels were significantly higher in diabetic groups as compared to control (P<0.05) which corresponded to a larger IS in diabetic rats relative to C (Fig. 1E). NAC and ALP conferred a synergistic effect in attenuating post-ischemic CK-MB release in diabetic rats. Pretreatment with either SnPP-IX or 2ME2 abolished the effect of NAC and ALP in reducing plasma CK-MB.

### Effect of NAC and ALP on Myocardial Injury and Function before and after MI/R

Apoptotic myocardial cell death was significantly increased in diabetic rats as evidenced by a larger number of TUNEL-staining positive cells (73.3±13.8% in group D vs. 5.6±1.2% in C, P<0.01). Four weeks treatment with NAC or ALP given alone significantly reduced the number of TUNEL-staining positive cardiomyocytes while their combination did not further decrease apoptotic myocardial cell death.

As shown in table 2, the echocardiography showed that the left ventricular (LVM/BM) was significantly elevated in the diabetic group compared to controls (P<0.01 vs. C), which was significantly attenuated by treatment with NAC and ALP. The heart rate in the diabetic rats as measured by echocardiography was lower than that in the control rats, and neither NAC nor ALP or their combination had significant impact on this parameter. The LV dimensions of the diabetic hearts were decreased manifested as reductions in LVIDd and LVVd (P<0.01 vs. C). These parameters were not significantly affected by NAC and/or ALP. The values of LVIDs and LVVs in diabetic rats did not significantly differ from that in controls. However, NAC and ALP significantly reduced LVVs (P<0.05, D+NA vs. D), resulting in significantly enhanced LVEF (P<0.05, D+NA vs. D). The SV in diabetic rats was significantly lower than that in the control group (P<0.05) and ALP but not NAC significantly enhanced SV to a level comparable to that in the control.

**Table 2:** M-mode echocardiographic and transmitral Doppler flow velocity indices of LV dimension and functions.

Parameters	C	D	D+N	D+A	D+NA	
HR (bpm)	317±13	281±23^a^	286±24^a^	273±29^a^	281±27^a^	
LVIDd (mm)	8.29±0.56	7.96±0.76^a^	7.82±0.29^a^	7.67±0.58	7.57±0.57^a^	
LVIDs (mm)	4.70±0.49	4.50±0.49	4.67±0.64	4.13±0.59^a^	4.10±0.34^a^	
LVVd (µl)	371.69±57.01	318.19±61.03^a^	319.44±45.2^a^	344.11±56.49	297.44±50.40^a^	
LVVs (µl)	107.83±26.11	92.647±24.78	101.60±32.59	76.90±25.23^ab^	73.54±21.89^ab^	
LVEF (%)	71.78±5.56	71.04±4.45	68.32±7.35	77.32±5.45^ab^	75.78±4.34^ab^	
IVRT (ms)	22.35±2.64	31.34±4.66	29.34±3.67	27.32±2.66	29.32±2.54	
E/A	1.50±0.12	1.20±0.03	1.54±0.11	1.67±0.23	1.76±0.43	
SV (µl)	263.87±43.09	225.38±42.67^a^	217.90±30.0	267.30±41.6^b^	223.66±36.47	
LVM (mg)	931.32±126.56	840.23±123.54^a^	704.45±91.6^ab^	768.32±85.65^ab^	703.34±118.65^ab^	
BW (g)	513.75±33.57	332.67±47.34^a^	336.77±31.4^s^	338.54±40.78^a^	310.23±26.67^a^	
LVM/BW (mg/g)	1.82±0.11	2.59±0.24^a^	2.32±0.54^s^	2.23±0.32^ab^	2.37±0.25^a^	

HR, heart rate; LV, left ventricle; LVIDd, LV internal diastolic diameter; LVIDs, LV internal systolic diameter; LVM, LV mass; LVVd, LV end-diastolic volume; LVVs, LV end-systolic volume; IVRT, isovolumetric relaxation time; SV, stroke volume; EF, ejection fraction. All values are means ±S. E.M, n = 8 per group.

a P<0.05 or P<0.01 compared to control groups,

b P<0.05 or P<0.01 compared to diabetes group.

**Figure 2:**
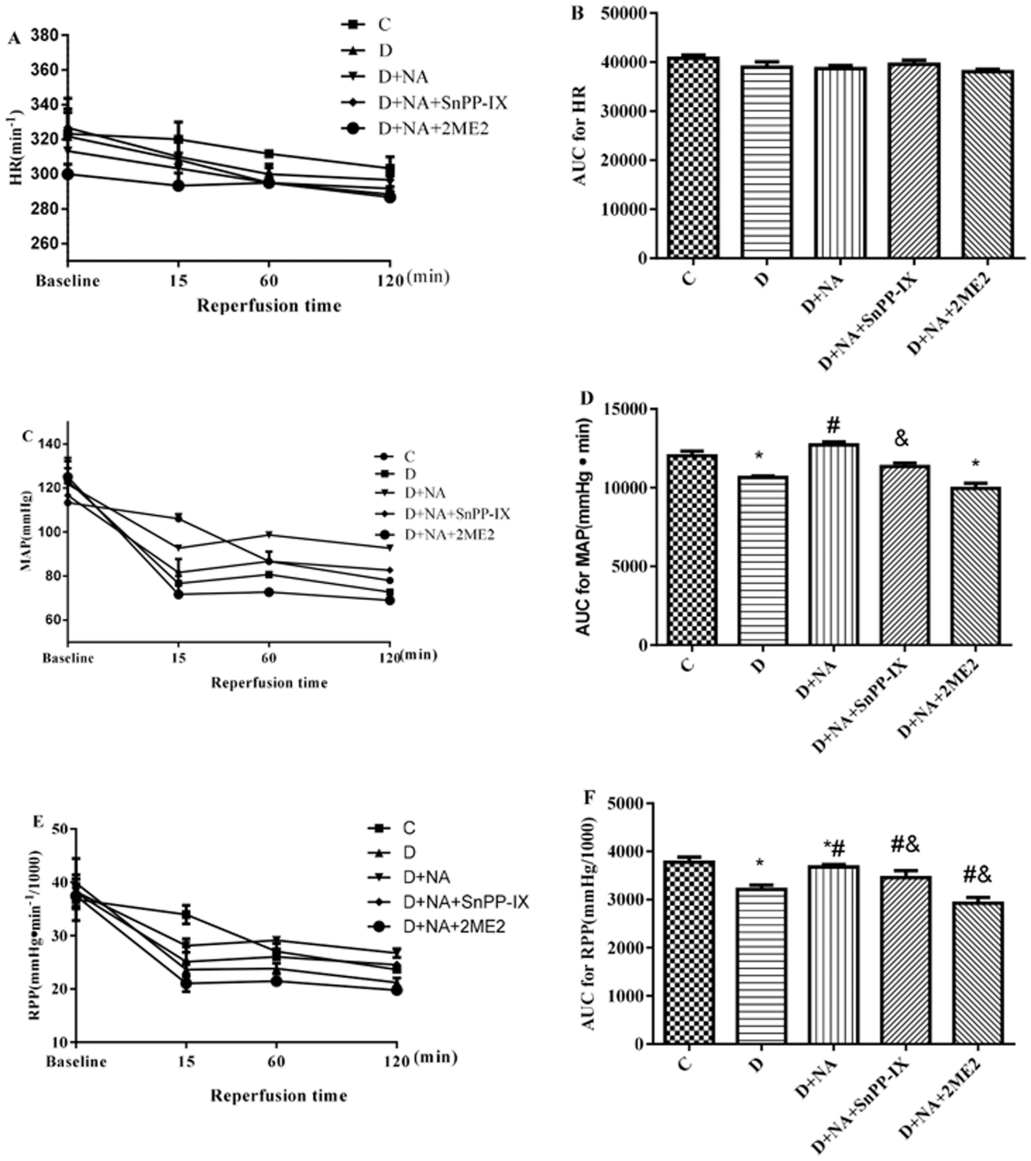
Hemodynamic changes measured at baseline (10 minutes before ischemia), at 15 minutes of ischemia, at 60 minutes of reperfusion, and at 120 minutes of reperfusion in diabetic rats with or without antioxidant treatment. Heart rate (HR), mean arterial pressure (MAP) and rate pressure product (RPP) were measured at baseline and during ischemia and their area under the curves (AUC) are shown. C and D indicate control or diabetes, respectively. D+NA, D+ NA+ SnPP-IX, D+NA+2ME2 indicates diabetic rats, treated with NAC+ALP (NA) or NA+SnPP-IX or NA +2ME2, respectively. All values are expressed as means ± S.E.M. (n = 7). ^*^P<0.05or P<0.01 vs. their corresponding baseline; ^#^P<0.05 or P<0.01 vs. their corresponding D groups; ^&^P<0.05 or P<0.01 vs. their corresponding D+NA groups.

During MI/R, heart rate did not significantly differ among groups over time (Fig. 2. A& B). Values for mean arterial blood pressure (MAP, a major hemodynamic parameter) and the product of heart rate and MAP (RPP) were relatively lower in diabetic rats compared to control rats at baseline (i.e., before inducing ischemia), but these difference did not reach statistical significance (Fig. 2 C & E). However, during ischemia-reperfusion, MAP and RPP were significantly lower in the diabetic group compared to control, and NAC and ALP normalized MAP and RPP (P>0.05 D+NA vs. C, P<0.05 D+NA vs. D). SnPP-IX and especially 2ME2 abrogated the effects of NAC and ALP in restoring normal values for MAP and RPP.

### Oxidative Stress and Antioxidant Status before and after Inducing MI/R

**Figure 3:**
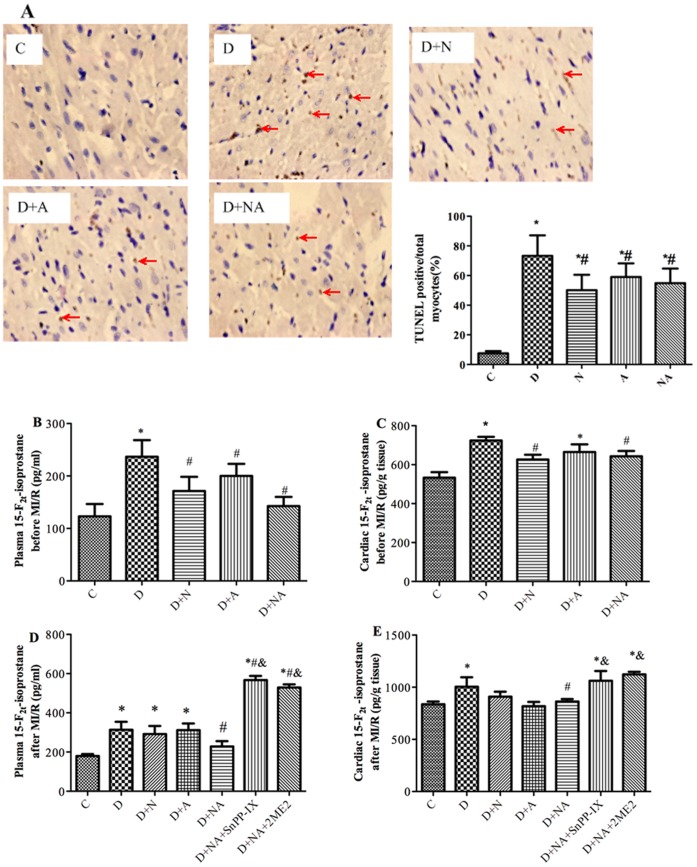
Effects of NAC and ALP on myocardial cell apoptosis assessed by TUNEL staining (A), and on cardiac and plasma 15-F2t-IsoP before and after myocardial ischemia/reperfusion (MI/R) (B–E). TUNEL positive cells were stained brown as indicated by arrow (A). Plasma and cardiac levels of 15-F2t-IsoP were assessed either after the completion of four weeks of NAC and ALP treatment (B, C) or after 30 minutes of ischemia and two hours of reperfusion in control and diabetic rats (D, E). Sham, C and D indicate sham operation, control or diabetes respectively. D+NA, D+ NA+SnPP-IX, D+NA+2ME2 indicate diabetic rats treated with NAC+ALP (NA) or NA +SnPP-IX or NA +2ME2, respectively. Data are expressed as means ±S.E.M. (n = 7). ^*^P<0.05 or P<0.01 vs. C; ^#^P<0.05 vs. D, ^&^P<0.05 vs. D+NA.

At baseline (i.e., before inducing ischemia) the plasma 15-F2t-IsoP concentration was significantly higher in diabetic rats than in controls, NAC and/or ALP normalized plasma 15-F2t-IsoP level in the diabetic animals (Fig. 3B). Similarly, cardiac tissue 15-F2t-IsoP was significantly increased in diabetic rats compared to controls and NAC and/or ALP significantly reduced cardiac 15-F2t-IsoP to a level comparable to that in the control group (Fig. 3C).

After two hours of post-ischemic reperfusion, plasma and cardiac 15-F2t-IsoP levels were significantly higher in diabetic than in control rats (Fig. 3D and E). The NAC and ALP combination treatment significantly reduced the plasma and cardiac 15-F2t-IsoP level to a level comparable to that in the control, while given alone the compounds did not significantly attenuate the increase of plasma 15-F2t-IsoP during reperfusion (Fig. 3D). HO-1 and HIF-1α inhibitors not only completely cancelled the effect of NAC and ALP in reducing the 15-F2t-IsoP level but further exacerbated post-ischemic oxidative stress as evidenced by the further elevated 15-F2t-IsoP content compared to the diabetic untreated group (All P<0.05, Fig. 3D and E).

**Figure 4:**
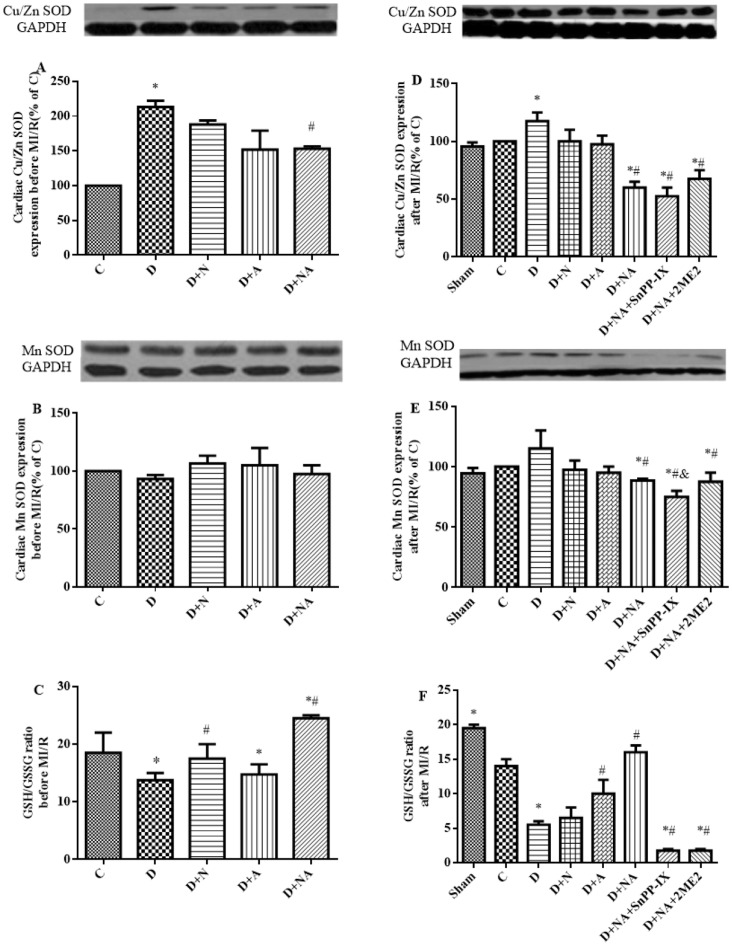
Effects of NAC and ALP on Cu/Zn superoxide dismutase (Cu/Zn SOD) (A and B), Mn SOD protein expression (D and E) before and after ischemia and reperfusion. Cardiac GSH/GSSG level before and after cardiac ischemia reperfusion(C and F).Sham, C and D indicate sham operation, control or diabetes respectively. D+NA, D+ NA +SnPP-IX, D+NA+2ME2 indicate diabetic rats treated with NAC+ALP (NA) or NA +SnPP-IX or NA +2ME2, respectively. Data are expressed as means ± S.E.M. (n = 7) ^*^P<0.05 vs. C; ^#^ P<0.05 vs. D.

At five weeks of diabetes, myocardial Cu/Zn SOD protein expression was significantly increased (P<0.05 vs. C) (P<0.01 vs. C, Fig. 4A). NAC or ALP partially, and their combination significantly attenuated this compensatory increase in Cu/Zn SOD protein expression. By contrast, myocardial Mn SOD protein did not significantly change in diabetic rats five weeks after STZ-injection and NAC and/or ALP treatment had no significant effect on this protein (Fig. 4B). However, Cu/Zn SOD expression significantly increased (P<0.05 vs. C Fig. 4D) while Mn SOD expression moderately increased (P>0.05 vs. C. Fig. 4E) in the diabetic group after MI/R. Both Cu/Zn SOD and Mn SOD expressions were significantly decreased after MI/R in rats treated with NAC and ALP (All P<0.05). The HIF-1α inhibitor 2ME2 but not the HO-1 inhibitor SnPP-IX prevented the significant decreases in myocardial Cu/Zn SOD and Mn SOD protein after MI/R following NAC and ALP treatment (Fig. 4D and E).

The cardiac GSH/GSSG ratio was significantly decreased in five-weeks diabetic rats compared to the control group (P<0.05 vs. C, Fig. 4C). NAC, but not ALP, restored the GSH/GSSG ratio. The combinational use of NAC and ALP not only restored GSH/GSSG ratio but further increased this ratio to a level higher than that in the control group (P<0.05 D+NA vs. C). MI/R resulted in dramatic reduction of GSH/GSSG ratio in the heart of diabetic rats as compared to control rats and NAC and ALP restored the GSH/GSSG ratio back to the control level (Fig. 4F). However, neither SnPP-IX nor 2ME2 were sufficient to abolish the synergistic effect of NAC and ALP in restoring GSH/GSSG.

### Effects of NAC and ALP on Myocardial HIF-1α/HO-1 Signaling in Diabetic Rats before and after MI/R

**Figure 5:**
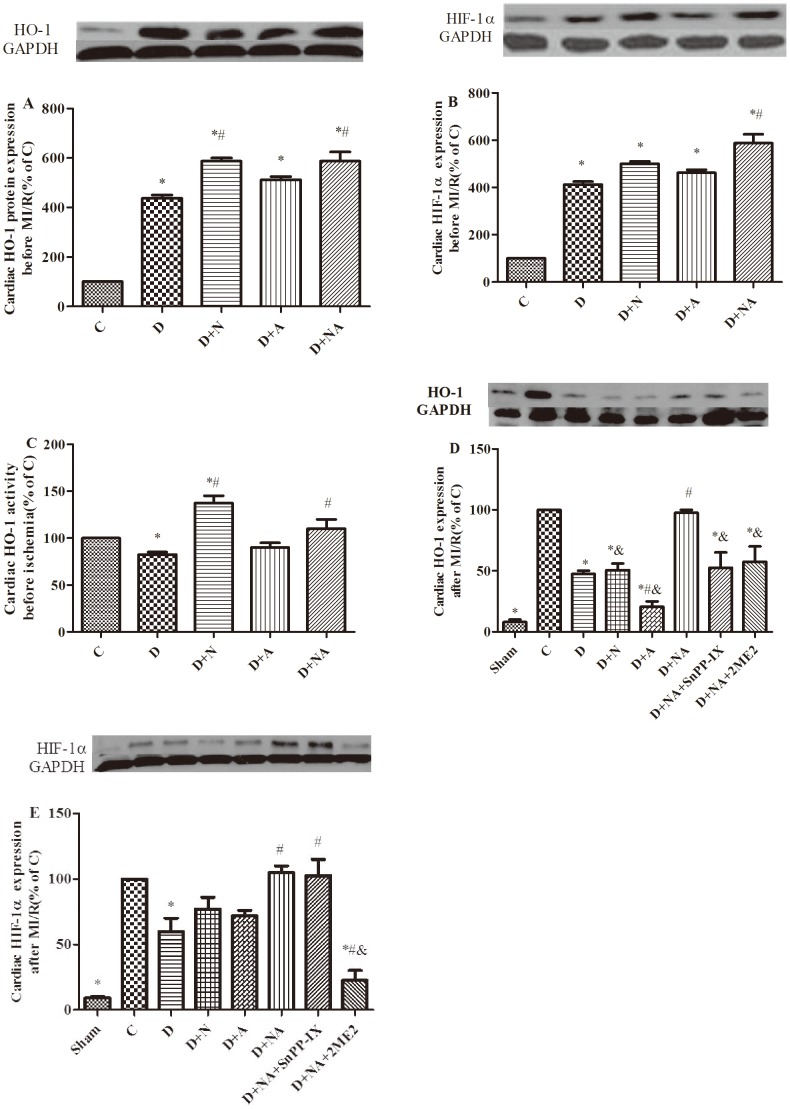
HIF-1α/HO-1 level changes before and after 30 minutes of ischemia followed by two hours of reperfusion after four weeks of NAC and ALP treatment. Myocardial HO-1 protein expression before (A) and after(C) MI/R. Myocardial HO-1 activity before(B) MI/R. HO-1activity was determined by measuring bilirubin formation, and calculated as percentage with respect to control. Myocardial HIF-1α protein expression before (D) and after (E) MI/R. Data are expressed as means± S.E.M. (n = 8). ^*^P<0.05 vs. C; ^#^P<0.05 or p<0.01 vs. D, ^&^P<0.01 vs. D+NA.

At five weeks of diabetes, myocardial HO-1 protein expression was augmented in diabetic rats relative to controls (Fig. 5A). NAC and the NAC/ALP combination but not ALP alone significantly further increased the myocardial HO-1 protein level compared to the untreated diabetic group (P<0.05, D+N or D+NA vs. D). Consistent with these changes in HO-1 protein, myocardial HO-1 activity was increased in diabetic rats (P<0.05 D vs. C, Fig. 5C) and NAC as well as the NAC plus ALP combination restored myocardial HO-1 activity (P<0.05 D+N or D+NA vs. D; P>0.05 D+N or D+NA vs. C, Fig. 5C).

After MI/R, myocardial HO-1 protein expression was dramatically reduced in diabetic rats compared to non-diabetic control (P<0.05, D vs. C; Fig. 5D). The NAC/ALP combination but not NAC or ALP alone increased post-ischemic myocardial HO-1 protein expression in diabetic rats to a level significantly higher than that in the untreated diabetic heart (P<0.05 D +NA vs. D), while SnPP-IX and 2ME2 abolished the effects of NAC/ALP on post-ischemic myocardial HO-1 protein (Fig. 5D).

Similar to the changes of myocardial HO-1 protein expression, myocardial HIF-1α protein expression was significantly increased in five-week diabetic rats (P<0.05 D vs. C, Fig. 5B). NAC/ALP significantly enhanced cardiac HIF-1α protein expression (P<0.05 D+NA vs. D; P>0.05 D+NA vs. C, Fig. 5B). After MI/R, myocardial HIF-1α protein was significantly lower in diabetic rats compared to control (Fig. 5E) and NAC/ALP combination but not NAC or ALP alone restored post-ischemic HIF-1α protein expression (P<0.05 D +NA vs. D; P>0.05 D +NA vs. C, Fig. 5E). 2ME2 not only completely cancelled the effects of NAC/ALP in restoring post-ischemic HIF-1α protein expression, but further reduced HIF-1α protein to a level lower than that in the diabetic untreated group (P<0.05 D+NA+2ME2 vs. D or D+NA, Fig. 5E). By contrast, SnPP-IX had no significant effect on HIF-1α expression compared with the diabetic untreated group.

### Changes of HIF-1α/HO-1 Signaling and Antioxidant Status in Cardiomyocytes Exposed to Hyperglycemia and H/R

**Figure 6:**
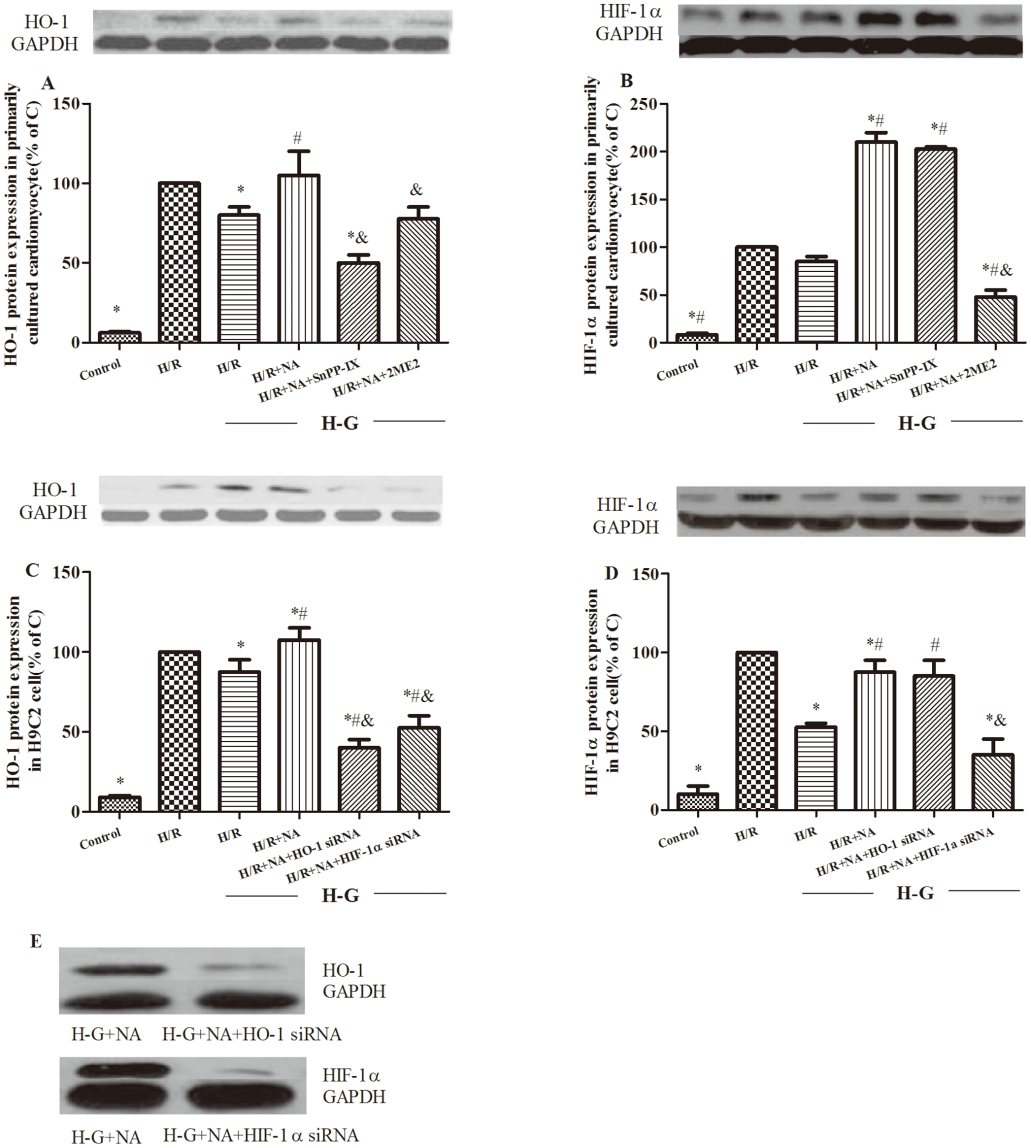
HO-1(A), HIF-1α (B) protein expression after 45 minutes of hypoxia followed by two hours of re-oxygenation in isolated rat cardiomyocytes under high glucose stimulation in the absence or presence of selective inhibitors. HO-1 siRNA and HIF-1α siRNA were applied, respectively, to knock down HO-1 and HIF-1α genes in H9c2 cell line and the effects were confirmed by assessing HO-1(C), HIF-1α (D) protein expression. Representative Western blots of HO-1 and HIF-1α gene knock down in H9C2 cells exposed to high glucose (E). Data are expressed as means± S.E.M. of two independent experiments each performed in triplicate. ^*^P<0.05 or P<0.01 vs. C; ^#^P<0.05 or P<0.01 vs. H-G, ^&^P<0.05, or P<0.01 vs. H-G+NA.

**Figure 7:**
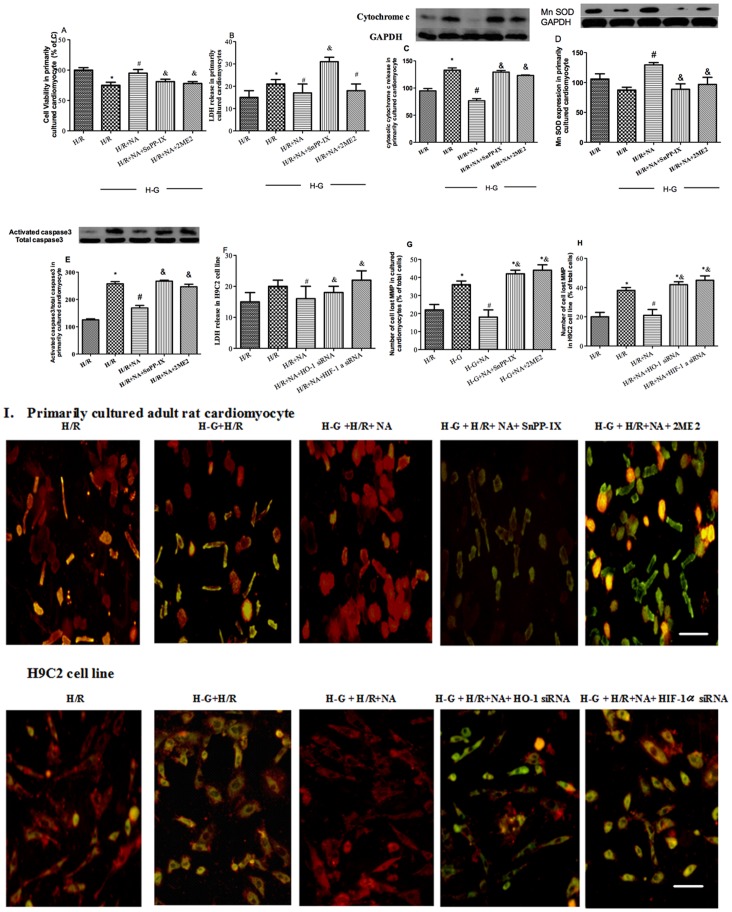
Cardiomyocyte injury after 45 minutes of hypoxia followed by two hours of re-oxygenation under high glucose stimulation. Effects of NAC/ALP on cell viability assessed by MTT assay (A), lactate dehydrogenase (LDH) release (B), Cytochrome c (C), Mn SOD (D) expression and the ratio of activated caspase3/total caspase3 (E) and mitochondrial membrane potential(MMP) loss (G) in presence or absence of HIF-1α/HO-1 signaling blockage in primary cultured cardiomyocytes. Effects of NAC/ALP on LDH release (F) and MMP loss in the presence or absence of HIF-1 α/HO-1 gene silencing in H9C2 cell line (H). MMP loss is expressed as percentage of cells positive for green fluorescence as quantified from microscopy images. Representative images of mitochondrial membrane potential loss in primarily cultured cardiomyocytes (I, upper lane) and H9C2 cell lines (I, lower lane). Isolated adult primary cardiomyocytes or H9C2 cell lines were plated at a density of 5×10^4^ cells/well with 48 h high glucose treatment. All cells were subjected to 45 minutes hypoxia and two hours of re-oxygenation with or without antioxidant treatment or with HIF-1α or HO-1 gene knockdown. Cells had strongest J-aggregation (red, 560/595 nm) due to high MMP and cells stained green (485/535 nm) had low MMP. Data are expressed as means± S.E.M. of two independent experiments each performed in triplicate (n = 6 per group).^ *^P<0.05 or P<0.01 vs. H/R; ^#^P<0.05 or P<0.01 vs. H-G+H/R, ^&^P<0.05, or P<0.01 vs. H-G+H/R+NA.

Following 45 minutes of hypoxia and 120 minutes of re-oxygenation, HO-1 protein expression was significantly lower in cardiomyocytes exposed to high than in those exposed to normal glucose (P<0.05, Fig. 6A) while HIF-1α expression did not significantly changed when cardiomyocytes were exposed to high glucose and H/R (P>0.05, H-G+H/R vs. H/R, Fig. 6B). Cell viability was significantly reduced (Fig. 7A) and cellular LDH activity increased (Fig. 7B) in cardiomyocytes exposed to high glucose. NAC and ALP normalized HO-1 protein expression in high-glucose treated cardiomyocytes and prevented H/R induced reduction of cell viability and increase in cellular LDH activity. However, NAC and ALP increased cardiomyocyte HIF-1α protein more than two fold (P<0.01 H-G+H/R+NA vs. H-G+H/R, Fig. 6B), and this effect of the agents was inhibited by 2ME2. SnPP-IX or 2ME2 also abolished the effects of NAC and ALP on post-hypoxic cardiomyocyte viability (Fig. 7A). SnPP-IX not only reversed the effect of NAC and ALP in reducing cardiomyocyte LDH release following H/R, but increased post-H/R LDH release to a level higher than that observed in the high glucose-untreated group (P<0.05 H-G+H/R+ NA+ SnPP-IX vs. H-G+H/R, Fig. 7B).

HIF-1α and HO-1 were knocked down prior to inducing H/R in H9C2 cell line. Transfection of H9C2 cells with HO-1 siRNA or HIF-1α siRNA decreased the corresponding protein expression by more than 90% (Fig. 6E). In line with the primary cardiomyocyte experiments, the HO-1 (Fig. 6C) and HIF-1α (Fig. 6D) protein expressions were decreased and LDH release was increased in H9C2 cells after exposure to high glucose and H/R as compared to the control group (Fig. 7F). NAC and ALP normalized the HIF-1α and HO-1 protein expression and LDH activity. The HIF-1α and HO-1 protein expressions were decreased after transfection with their respective siRNA. HIF-1α or HO-1 signaling knockdown significantly decreased HIF-1α and HO-1 protein expression and resulted in increased post-H/R LDH activity (P<0.05 H-G+H/R+NA+HIF-1α siRNA or H-G+H/R+NA+HO-1 siRNA vs. H-G+H/R or H-G+H/R +NA, Fig. 6 C, D and Fig. 7F).

### Cardiomyocyte Mitochondrial Membrane Potential (MMP) and Cell Injury

After H/R, the MMP loss in normal glucose treated cardiomyocytes was about 22%, while in cardiomyocytes exposed to high glucose, the percentage of loss was increased significantly (P<0.05, H-G-H/R vs. H/R. Fig. 7G). This was accompanied by significantly increases in cytosolic cytochrome c release (Fig. 7C) and caspase3 activation (Fig. 7E) but by a significant reduction in Mn SOD (Fig. 7D) as compared to normal glucose control (all P<0.05). NAC and ALP treatment prevented high-glucose and H/R mediated exacerbation of MMP loss increases in caspase3 activation and cytosolic cytochrome c release and as well as it restored Mn SOD protein expression. These beneficial effects of joint antioxidant NAC/ALP treatment were abolished by either SnPP-IX or 2ME2.

Similarly, in cultured H9C2 cells, either HIF-1α or HO-1 gene knockdown abrogated the effects of NAC/ALP in attenuating high glucose and H/R induced increases in MMP (Fig. 7H) and cellular LDH release (Fig. 7F).

## Discussion

The present study shows that cardiac HIF-1α and HO-1 protein expressions are reduced in diabetic hearts *in vivo* and in cultured cardiomyocytes exposed *in vitro* to high glucose. These changes are concomitant with increased oxidative stress and exacerbated cardiomyocyte cellular injury after ischemic/hypoxic stimulation. NAC and ALP synergistically attenuate post-ischemic injury and cardiac dysfunction in type 1 diabetic rats and in isolated cultured adult rat cardiomyocytes exposed to high glucose and H/R, an effect associated with enhancement of cardiac HIF-1α/HO-1 signaling in response to MI/R. The subsequent attenuation of post-ischemic oxidative injury and the protective effects of NAC and ALP were cancelled by inhibition of either HIF-1α or HO-1. Similarly, in cultured adult rat cardiomyocytes exposed to high glucose, the NAC and ALP exerted post-hypoxic cellular protection was associated with enhancement of HIF-1α and HO-1 protein expression. Indeed, inhibition of HIF-1α or HO-1 or their gene knockdown abrogated the protective effects of the antioxidants. Thus, the present findings indicate that the cardioprotective effects in diabetes resulting from the combination of NAC plus ALP were achieved primarily through the restoration of HIF-1α/HO-1 signaling in response to MI/R.

CK-MB, the sensitive cardiac injury marker, was significantly higher in diabetic heart than that in the control rat after 30 minutes of ischemia and two hours of reperfusion, which indicated that post-ischemic myocardial cellular injury is more severe in diabetic than that in the control rats. Infarct size is viewed as the gold standard in assessing the severity of post-ischemic myocardial injury, while CK-MB is a reliable predictor of post-ischemic myocardial infarction in patients after acute myocardial ischemia [45], [46]. In patients with acute myocardial ischemia, the plasma CK-MB level peaks about ten hours after ischemia and predicts well myocardial infract size (IS) five to seven days after reperfusion with thrombolytic therapy [46]. Plasma CK-MB levels correlate inversely with post-ischemic left ventricular function and myocardial infarction five to ten days after an ischemic episode [31]. Similarly, previous findings in isolated ischemic reperfused rat hearts showed that the increase in post-ischemic CK-MB peaked minutes after reperfusion while significant myocardial IS did not become apparent until after one hour of reperfusion [47]. In the present study, significantly higher post-ischemic CK-MB levels in diabetic rats at reperfusion correlated with more severely impaired post-ischemic left ventricular function as compared to controls. The increase (compared with controls) in post-ischemic IS in the diabetic rats in the present study did not reach statistical significance despite the significant increase in CK-MB level. A possible reason may be that the reperfusion time of 120 minutes was not long enough to catch significant increase in IS in the diabetic group when using 30 minutes of coronary ligation model, given that an *in vivo* study in mice shows that post-ischemic myocardial IS progressively increases when reperfusion time varies from 120 minutes to 240 minutes after 30 to 40 minutes of coronary artery occlusion [48]. Significantly higher levels of CK-MB and more severe impairment in post-ischemic left ventricular function in the diabetic rats as shown in the present study indicate that myocardial injury is more severe in diabetic than in control rats. The antioxidants NAC and ALP achieve synergy in enhancing endogenous antioxidant capacity and most profoundly attenuate post-ischemic MI/R injury in diabetes.

Earlier observation [49] demonstrated that NAC treatment alone attenuated but did not prevent myocardial dysfunction in diabetic rats. Similarly, experimental antioxidant treatment with single agents has been shown to attenuate but not to prevent myocardial ischemia-reperfusion injury [50], [51] and cardiac dysfunction both in normal and in diabetic subjects [52], [53]. In the present study, NAC and ALP in combination, but neither one of them alone as shown in a previous study [22] normalized post-ischemic mean arterial blood pressure in diabetic rats and significantly reduced post-ischemic myocardial infarction, which may be explained in part through additional effects resulting from the different antioxidant properties of the two agents.

ALP, a xanthine oxidase inhibitor, attenuates diabetes-induced ROS production, and unlike NAC it also improves diabetes-induced cardiac dysfunction [54]. Xanthine oxidase, a source of ROS, is increased in the ischemic tissue as a result of enhanced conversion of xanthine dehydrogenase to xanthine oxidase [55], which increases oxidative stress and exacerbates tissue ischemic injury [36]. Increased xanthine oxidase/hypoxanthine activity prompts opening of cardiac mitochondrial permeability transition pore (MPTP) and exacerbates cardiomyocyte injury [56]. Therefore, combating ROS-induced cellular damage and preventing MPTP opening by enhancing GSH/GSSG (the largest capacity thiol buffer in the cell, the reduction of which is one of the major mechanisms that prompt the opening of MPTP in diabetes [57]) and inhibiting xanthine oxidase could be the fundamental mechanism explaining why NAC and ALP attenuate the augmented myocardial post-ischemic injury in diabetic rats and ameliorate post-hypoxic cardiomyocyte injury under high glucose stimulation. Oxidative stress was increased under diabetic conditions and accompanied by the development of diabetic cardiomyopathy, with apoptosis and fibrosis as early features of heart failure. ALP, as shown in a previous study [30] and the study of Rajesh and colleagues [58] significantly attenuated the development of cardiac fibrosis and dysfunction in diabetic rodents. Further, the finding of the present study that ALP significantly reduced myocardial apoptosis and caspase3 activation in diabetic rats is in keeping with the findings of Rajesh and colleagues [58] who showed that ALP treatment, in addition to reducing oxidative and nitrative stress, reduced myocardial apoptotic cell death in a mouse model of STZ-induced type 1 diabetes. These properties of ALP can be attributed to the NAC/ALP synergistic myocardial protection demonstrated in the current study. During the combined NAC and ALP treatment, the improvement of left ventricular function prior to inducing myocardial ischemia as shown by echocardiograph study should also have contributed in part to the attenuation of post-ischemic myocardial injury in the diabetic animals.

NAC, a precursor of GSH, conferred its antioxidant effect in the diabetic myocardium primarily via enhancing endogenous GSH/GSSG but not via myocardial SOD. That NAC/ALP treatment can enhance endogenous GSH/GSSG to a level higher than observed in control rats at basal may explain why chronic NAC/ALP treatment prevented the compensatory increase in myocardial Cu/Zn SOD in diabetes or even reduced cardiac Cu/Zn SOD and Mn SOD after ischemia, but still decreased cardiac and plasma 15-F2t-Isop to levels comparable to those in the control group and also reduced post-ischemic myocardial infarction in the diabetic rats.

In addition, the cardioprotective effects of the NAC and ALP combination are associated with attenuation of the loss of cardiomyocyte MMP after exposure to high glucose and hypoxia/re-oxygenation. Mitochondria are both a major source of ROS and also a critical target during oxidative damage. Therefore, mitochondrial oxidative stress-induced dysfunction has been implicated in the pathogenesis of many diseases such as diabetes and ischemia/reperfusion injury. Cytochrome c release from mitochondria and MMP loss (which leads to the increase in MPTP opening) are critical events resulting in cell apoptosis [59]. Reduction in Mn SOD and the subsequent increase in mitochondrial oxidative stress play important roles in this pathology. The present study shows that NAC/ALP attenuated the increased release of cytosolic cytochrome c and the decrease of Mn SOD and MMP through mechanisms that involve HIF-1α/HO-1 signaling. The novel finding that NAC and ALP attenuate high glucose-induced MPTP opening in cardiomyocytes during hypoxia/re-oxygenation via HIF-1α/HO-1 signaling may have potential clinical importance in guiding the development of effective therapies to combat myocardial ischemia in diabetes. Indeed, mitochondria from diabetic hearts are more sensitized to MPTP opening than those from non-diabetic hearts as a result of redox-dependent shifts in MPTP opening [57]. The latter could be the major reason why diabetic hearts are more vulnerable to ischemic insult.

The antioxidants NAC and ALP, at doses that can normalize the levels of the specific oxidative stress index 15-F2t-IsoP, stabilized cardiac HIF-1α, moderately enhanced HIF-1α protein expression in the diabetic myocardium, and subsequently attenuated post-ischemic myocardial injury. Of particular interest, NAC and ALP acted synergistically to enhance myocardial HIF-1α protein expression and most prominently reduced post-ischemic myocardial injury. This synergistic cardioprotection exerted by NAC and ALP in a model of myocardial ischemia-reperfusion injury in diabetic animals was prevented by *in vivo* administration of either HO-1 or HIF-1α inhibitors. Likewise, *in vitro* knockdown of HO-1 or HIF-1α to prevent NAC/ALP mediated enhancement of HO-1 or HIF-1α, suppressed the protection exerted by the antioxidants against cardiomyocyte post-hypoxic cellular injury and was accompanied by a loss of cell MMP. Thus, the results from the present study indicate that activation of cardiac HIF-1α and its downstream molecular target HO-1 play a key role in antioxidant cardioprotection in the diabetic heart.

The cardiac HIF-1 α/HO-1 levels were increased in response to I/R or H/R stimulation compared to control while the post-ischemic cardiac HIF-1 α/HO-1 levels were decreased both in diabetic rats and in isolated cardiomyocytes under high glucose conditions. This is consistent with and extends the finding of Marfella and colleagues who showed that HIF-1 α activation played a critical in maintaining myocardial viability in diabetic mice [8]. Indeed, HIF-1 α/HO-1 signaling plays an important role in the cardiovascular system [60]. Taken in conjunction, the findings by others and the present results suggest that the HIF-1 α/HO-1 pathway plays an essential role in cardioprotection against MI/R injury in diabetes, although the detailed regulation of the involvement of HIF-1α/HO-1 signaling warrants further investigation.

In summary, the present study demonstrates that NAC and ALP act synergistically in reducing MI/R injury in diabetes primarily by restoration of the HIF-1 α/HO-1 signaling. These results provide insight into the effects and mechanisms of action of antioxidants therapy that may lead to the development of effective therapeutic regimens to combat the myocardial complications of diabetes.
